# Cloning of a new glutathione peroxidase gene from tea plant (*Camellia sinensis*) and expression analysis under biotic and abiotic stresses

**DOI:** 10.1186/1999-3110-55-7

**Published:** 2014-01-18

**Authors:** Jian-Yu Fu

**Affiliations:** Tea Research Institute, Chinese Academy of Agricultural Sciences, 9 South Meiling Road, Hangzhou, 310008 China

**Keywords:** Abiotic stresses, Biotic treatment, Glutathione peroxidase, Induced, Tea plant

## Abstract

**Background:**

Tea plant, Camellia sinensis (L.) O. Kuntze, a well-known heavy metal hyperaccumulator, possesses a powerful tolerance to heavy metals. The heavy metal stresses lead to reactive oxygen species (ROS) production, and high concentration of ROS is harmful to plants. The glutathione peroxidase gene has positive function to damage induced by ROS. To understand the mechanism of tolerance to deferent stresses in tea plant, a new glutathione peroxidase gene of tea plant was cloned and its expression pattern was analyzed under abiotic and biotic stresses.

**Results:**

A novel cDNA encoding glutathione peroxidase of tea plant (Camellia sinensis) was isolated by rapid amplification of cDNA ends (RACE) method and designated as CsGPX2 (GenBank Accession No. JQ247186). This full-length sequence was 917 nucleotides including a 510 bp open reading frame (ORF), which encoded a polypeptide of 169 amino acids. The deduced amino acid sequence showed high homology with glutathione peroxidases of angiosperms and contained the characteristic conserved motifs of ILAFPCNQF and FTVKD, the highest level of similarity was 85% to a glutathione peroxidase from Ricinus communis (Accession NO. XP_002509790.1). Tissue expression pattern analysis indicated that CsGPX2 expressed similarly in root, stem, leaf and flower of tea plant. The CsGPX2 gene showed strong responses to most abiotic stresses including salinity, heavy metal toxicity, drought, heat, plant hormones, but could not be induced by biotic treatment.

**Conclusions:**

The result suggested that CsGPX2 had potential function in protecting tea plant from peroxidative damage induced by some abiotic stresses.

**Electronic supplementary material:**

The online version of this article (doi:10.1186/1999-3110-55-7) contains supplementary material, which is available to authorized users.

## Background

In plants, aerobic reactions lead to reactive oxygen species (ROS) production such as superoxide radical (·O_2_^-^), hydroxyl radical (·OH), and hydrogen peroxide (H_2_O_2_). It has been proved that ROS has two entirely different roles in plants, signal function and oxidative damage. When the ROS concentration is at an acceptable low level, they play an important signaling function in plants controlling processes such as growth, development, response to biotic and abiotic environmental stimuli, and programmed cell death (Bailey-Serres and Mittler 2006. While high concentration of ROS are harmful to cells (Rodríguez Milla et al. 2003; Navrot et al. 2006; Ramos et al. 2009), and consistent accumulation of ROS imposes ultimately oxidative stress, exacerbating cellular damages (Bhattacharjee 2012).

Under biotic or abiotic stress, ROS may dramatically accumulate in plants and generate oxidative damage (Faltin et al. 2010; Gill and Tuteja 2010; Suzuki et al. 2011). On the other side, plants have developed some enzymatic systems including superoxide dismutases (SOD), catalases (CAT), ascorbate peroxidases (APX), peroxiredoxins and non-enzymatic mechanisms to protect against oxidative damage caused by these ROS (Agrawal et al. 2002; Navrot et al. 2006; Anjum et al. 2012). Glutathione peroxidases (GPXs) catalyze the reduction of H_2_O_2_, organic hydroperoxides, and lipid peroxides using GSH and/or other reducing equivalents (Ursini et al. 1995).

The study of GPXs in plants followed the previous research in mammals (Criqui et al. 1992; Bae et al. 2009). Since the first plant GPX cDNA was isolated from a wild tobacco (*Nicotiana sylvestris*) (Criqui et al. 1992), a significant part of novel genes for GPXs were reported in succession. To date, more than 100 GPXs were isolated from diverse plants and even 8 GPXs in *Arabidopsis* were cloned (Gaber et al. 2012). These GPX genes from plants were clustered in five main groups (Holland et al. 1993; Jung et al. 2002). Clades I and II are hypothesized to contain, respectively, chloroplastic and cytosolic isoforms; clades III and IV, both cytosolic and secreted proteins; and clade V, cytosolic proteins and proteins with N terminal transit peptides for targeting either to the mitochondria or to both the mitochondria and chloroplasts (Margis et al. 2008; Ramos et al. 2009). Most of the plant GPXs show high similarity to animal phospholipid hydroperoxide glutathione peroxidases (PHGPXs) (Rodríguez Milla et al. 2003), but their structure, substrate specificities, and subcellular localization were large different with mammal GPXs (Miao et al. 2005; Miao et al. 2006; Yang et al. 2006).

Tea, *Camellia sinensis* (L.) O. Kuntze, originated in China, is one perennial woody evergreen plant. Tea plant, a heavy metal hyperaccumulator, cumulates heavy metals by uptake of them from soil and air consistently. Meanwhile, it possesses a corresponding tolerance to heavy metals (Feng et al. 2009; Anjum et al. 2012; Hossain et al. 2012). To date there is only one glutathione peroxidase gene (GenBank Accession No. AEC10977) was isolated from tea plant but no function research was involved. In the present study, another new glutathione peroxidase gene of tea plant was cloned and expression pattern was analyzed under simulated environmental conditions, plant hormones, herbivore damage. The result showed that the gene was induced by all abiotic stresses dramatically, but the gene was not sensitive to biotic treatment, and these findings may help us to understand the mechanism of tolerance to deferent stresses in tea plant.

## Methods

### Plants materials

Tea plants, *Camellia sinensis.* cv. Longjing 43 were cultured in vermiculite and kept in light incubator under controlled conditions (25°C and 10/14 h light/dark photoperiods) with 85% relative air humidity. Three-week-old seedlings were treated with all biotic and abiotic stresses, the harvested organs (roots, stems and leaves were collected from seedlings, flowers were picked from flowering field plants) were immediately frozen in liquid nitrogen and stored at -80°C until nucleic acid were extracted. For heavy metals, salinity and drought stresses, roots of 10 intact plants were partly soaked in 200 μM FeSO_4_, 200 μM CuSO_4_, 500 mM NaCl and 500 mM mannitol for 6 hours (Rodríguez Milla et al. 2003; Miao et al. 2006). For heat treatment, seedlings were kept under 40°C in chambers for 3 hours. For plant hormones treatments, seedlings were sprayed 2–3 times in 12 hours with 1 mM SA (salicylic acid), ABA (abscissic acid), GA (gibberellin), NAA (naphthaleneacetic acid) and MeJA (Methyl jasmonate) (Sigma-Aldrich, St Louis, MO, USA) solutions under continuous light (Navrot et al. 2006; Rodríguez Milla et al. 2003; Miao et al. 2006). Control seedlings were treated with deionized water. MeJA, GA and NAA was dissolved in sterilized water with 2% ethanol, SA and ABA were dissolved in sterilized water at an ultimate concentration of 1 mM. For biotic treatments, two larvae of tea geometrids (*Ectropis obliqua* Prout) starved for 24 h were placed on the foliage for feeding damage, and the damaged leaves were harvested after 6, 12, 24, 48, 72 and 96 hours. Leaves of intact plants were collected as control at the time the treatments started.

### The cDNA cloning and sequence analysis

Total RNA were isolated with a polysaccharide and polyphenol total RNA isolation kit (BioTeke, Beijing, China). The quality and concentration of the RNA were checked by NanoDrop 1000 spectrophotometer (Thermo Fisher Scientific, Waltham, MA, USA) and formaldehyde agarose gel electrophoresis. Total RNA was reverse transcribed to the first-strand cDNA with an oligo (dT) primer designed with an adaptor sequence according to the protocol of the SMART RACE cDNA Amplification Kit (Clontech, Mountain View, CA, USA.). The RACE PCR primers was designed and synthesized based on the sequence obtained from a cDNA library (a lab internal source) of tea plants. Primers of *Cs* GPX2-5′RACE (5′-ATCTGTTCACGAGTTCACCGTCAAGG-3′) and universal primer A mix (UPM, long: 5′-CTAATACGACTCACTATAGGGCAAGCAGTGGTATCAACGCAGAGT-3′; short: 5′-CTAATACGACTCACTATAGGGC-3′) were used to carry out the 5′RACE-PCR under the recommended condition by the kit. The 3′RACE-PCR were performed with the 3′-RACE CDS Primer A (5′-AAGCAGTGGTATCAACGCAGAGTAC(T)30 N_-1_ N-3′ and specific primer *Cs* GPX2*-* 3′RACE:5′-GGTGGATTTTTTGGTGATGGAA-3′) under the same condition as above-mentioned. The PCR products was purified by kit and subcloned into the pGEM-T Easy vector (Promega Corporation, Madison, WI, USA), transferred into *E. coli* DH5α and sequenced bidirectionally by ABI 3730 automated sequencer (Applied Biosystems, Foster City, CA, USA) with the universal primers of M13.

The two RACE products and original fragment were automatically aligned and assembled by DNAMAN program, and the full-length cDNA sequence of *Cs* GPX2 gene was obtained by splicing. The deduced amino acid sequence comparison was performed via BLAST program (NCBI, National Center for Biotechnology Services, http://www.ncbi.nlm.nih.gov). The *Cs* GPX2 and other plants glutathione peroxidase genes retrieved from GenBank were aligned with online CLUSTAL W (http://www.ebi.ac.uk/Tools/msa/clustalw2). The phylogenetic tree was constructed by MEGA 4 program based on the converted result from CLUSTAL W alignments.

### Expression analysis in organs and under different stresses

The *Cs* GPX_2_ expression profiles in different organs (roots, stems, leaves and flowers) of tea plant and under biotic and abiotic treatments were investigated by real time qRT-PCR. Total RNA were isolated from 100 mg of roots, stems, leaves, petals and treated leaves and reverse transcribed to the first-strand cDNAs with an oligo (dT) primer according to the manual of PrimeScript® RT reagent Kit (TaKaRa, Japan). The qRT-PCR was performed on an ABI 7500 Real-Time PCR System with the primers of *Cs* GPX2*-* F (5′-CCAGGAGCCAGGGAATAATGAG-3′) and *Cs* GPX2*-* R (5′-GGAGCAGCATTCTCACCATTCA-3′). The 18SrRNA gene (18Sr RNA-F: 5′-CGGCTACCACATCCAAGGAA-3′, 18Sr RNA-R: 5′-GCTGGAATTACCGCGGCT-3′) was used as internal control gene in all qRT-PCR reactions above (Sun et al. 2010).

## Results and discussion

### Cloning of full-length cDNA

Two fragments, a 286 bp long 3′- flanking region with poly (A) and a 519 bp long 5′- untranslated region (UTR) were obtained by RACE method respectively. Both sequences showed high homology with glutathione peroxidase genes of angiosperms, and they were spliced to get full-length cDNA. The complete nucleotide sequence was denominated as tea plant glutathione peroxidase 2 (*Cs* GPX2, Accession NO. JQ247186), because of an already known glutathione peroxidase gene named as *Cs*. GPX and according to the nomenclature used by Mullineaux *et al*^1998^; and Rodríguez Milla et al. 2003. The *Cs* GPX2 gene was 917 bp long including a 5′- UTR of 265 bp and a 3′- UTR of 142 bp, and the ORF was 510 bp with 169 deduced amino acids. The deduced amino acid sequence of *Cs* GPX2 (CsGPX2) contained the motif of ILAFPCNQF and FTVKD, which were commonly conserved in glutathione peroxidase family. Using the online Computer pI/Mw Tool http://cn.expasy.org/tools), the theoretical isoelectric point (*pI*) and molecular weight (MW) of *Cs* GPX2 was calculated, and the two values were 7.62, 18.8 kDa respectively.

### Sequence analysis and expression profiles in tea plant organs

**Figure 1:**
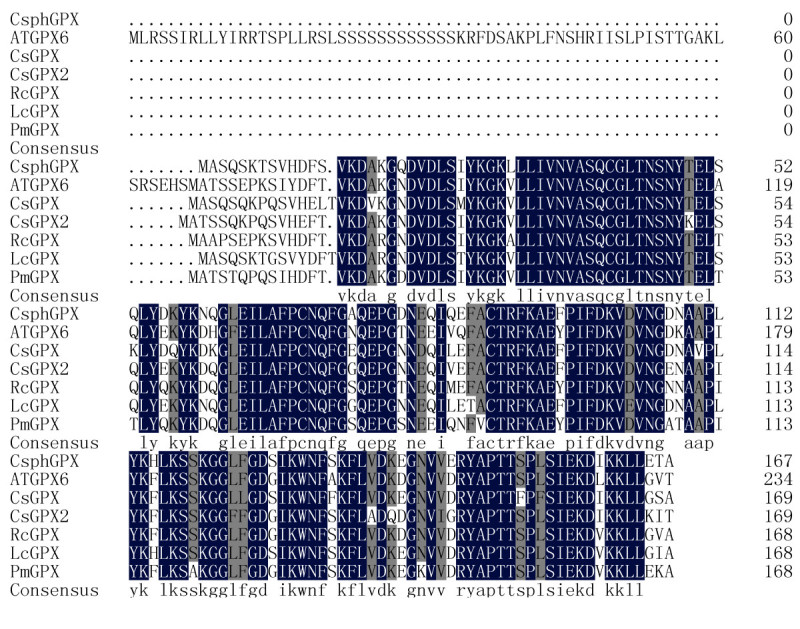
**CsGPX2 (this work) sequence was aligned with several glutathione peroxidases.** *Camellia sinensi* s, CsGPX2; *Ricinus communis*, RcGPX, XP_002509790; *Plantago major*, PmGPX, CAJ43709; *Litchi chinensis*, LcGPX, ACI04528; *Camellia sinensi* s, CsGPX, AEC10977, *Citrus sinensi* s, CsphGPX, CAE46896; and *Arabidopsis lyrata subsp. lyrata*, ATGPX6, XP_002874703.1 were aligned. Conserved sequence elements are highlighted and identical residues were showed in the consensus line. Shading indicates levels of sequence conservation (100% conservation: white on black; 75% conservation: black on dark grey).

**Figure 2:**
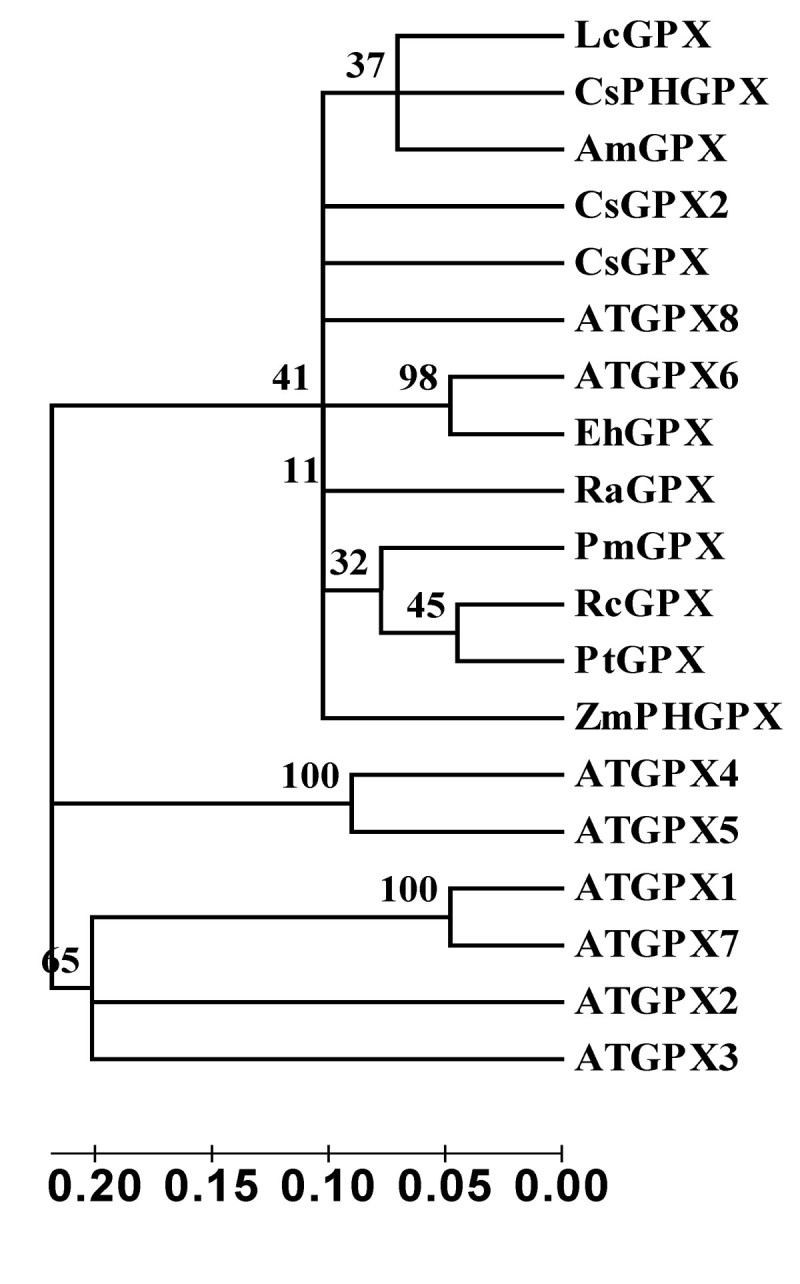
**The phylogenetic tree was constructed via NJ (Neighbor Joining) method based on amino acid sequences of glutathione peroxidases.** The scale was at the bottom, and the bootstrap values were shown above each branch. Accession numbers: CsGPX2, JQ247186; *Ricinus communis*, XP_002509790.1; *Plantago major*, CAJ43709; *Litchi chinensis*, ACI04528; *Camellia sinensis*, AEC10977; *Citrus sinensis*, CAE46896; *Arabidopsis lyrata subsp. lyrata*, XP_002874703.1; *Arabidopsis lyrata subsp. lyrata*, EFH55083.1; *Arabidopsis lyrata subsp. lyrata*, EFH57440.1; *Arabidopsis lyrata subsp. lyrata*, EFH58156.1; *Arabidopsis thaliana*, AEC10946.1; *Arabidopsis lyrata subsp. lyrata*, EFH52965.1; *Arabidopsis thaliana*, AEE85970.1; *Arabidopsis thaliana*, AEE34102.1; *Zea mays*, ACG39625.1; *Ammopiptanthus mongolicus*, AFC01207.1; *Eutrema halophilum*, ACP28875.1; *Rheum austral*, ACH63236.1; *Populus trichocarpa*, XP_002299536.1.

The deduced amino acid sequence of *Cs* GPX2 comparison was performed by Blast P online (http://www.ncbi.nlm.nih.gov/). The protein sequence was most similar to glutathione peroxidases of angiosperms with a similarity of 85% to glutathione peroxidases from *Ricinus communis*, *Arabidopsis lyrata subsp. lyrata*, *Populus trichocarpa* (Accession NO. XP_002509790.1, XP_002874703.1, XP_002299536.1). While the similarity (84%) with the known glutathione peroxidase gene of tea plant (Accession NO.AEC10977.1) was not as high as that of several genes above mentioned, which indicated that the two glutathione peroxidase genes *Cs* GPX and *Cs* GPX2 belonged to deferent clades (Figure 1). The sequence also showed high homologous with other characteristic conserved elements of glutathione peroxidases, including the most highly conserved metal ion-binding motif of ILAFPCNQF. A phylogenetic tree was constructed based on the *Cs* GPX2 sequence and other plant GPXs via CLUSTAL W2 online (http://www.ebi.ac.uk/) and MEGA 4 software. The evolutionary relationship between *Cs* GPX2 and selected sequences of higher plants was performed via NJ (Neighbor Joining) method, and the bootstrap values (1000 replicates) were shown above each branch (Figure 2). The phylogenetic tree clearly showed that GPXs from plants clustered in three groups, most GPXs including two gene of *Cs* GPX2 and *Cs* GPX from tea plant clustered one large group first, which suggested their divergence were recent with scales from 0.05 to 0.10. Two *Arabidopsis* GPXs of ATGPX4 and ATGPX5 clustered a separate group, which indicated the evolution of these proteins within it was independent. Four genes of ATGPX1, ATGPX2, ATGPX3 and ATGPX7 clustered another group, and the divergence scales between the other two groups were both larger than 0.20 respectively.

**Figure 3:**
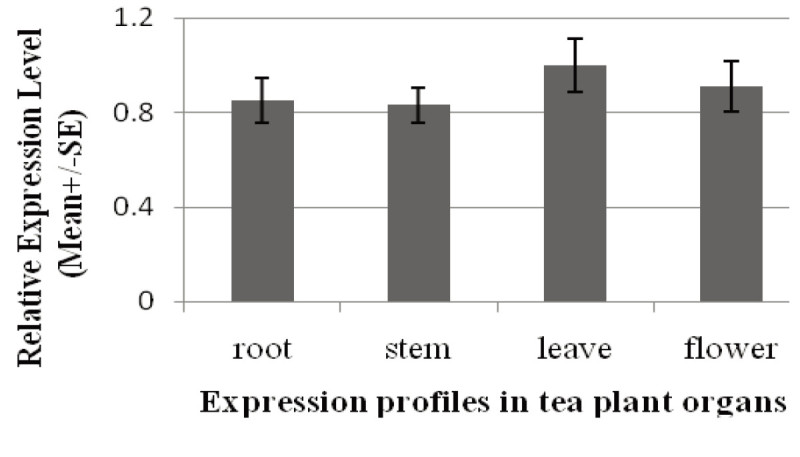
**The relative expression levels of** ***Cs*** **GPX2 in tea plant (** ***Camellia sinensis*** **) organs.** Gene transcript in root, stem, leaf and flower were detected by qRT-PCR.

The *Cs* GPX2 expression levels in deferent organs of tea plant were investigated by real-time qRT-PCR. The relative expression level was 0.85, 0.83, 1.00 and 0.91 in root, stem, leaf and flower respectively. This result showed that *Cs* GPX2 expressed at equivalent levels with no obvious difference in organs tested (Figure 3).

### *Cs* GPX2 expression pattern under biotic and abiotic stresses

**Figure 4:**
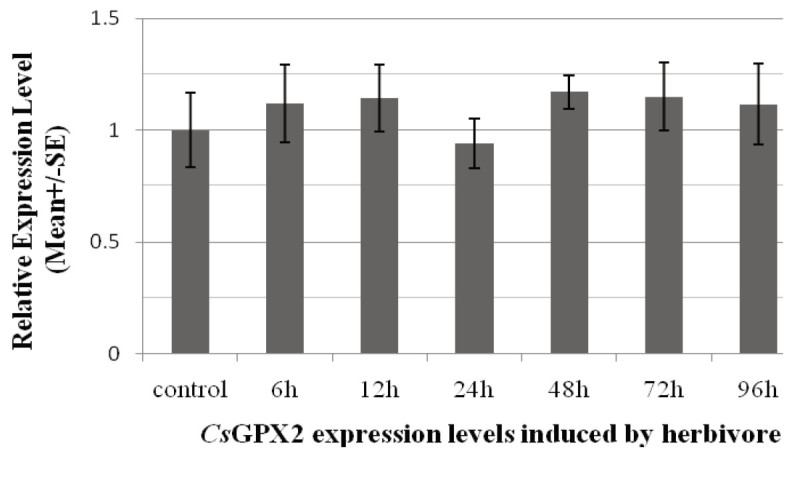
**The transcripts levels of** ***Cs*** **GPX2 induced by herbivore.** Gene expression levels were investigated by qRT-PCR after leaves were damaged for 6, 12, 24, 48, 72 and 96 h.

**Figure 5:**
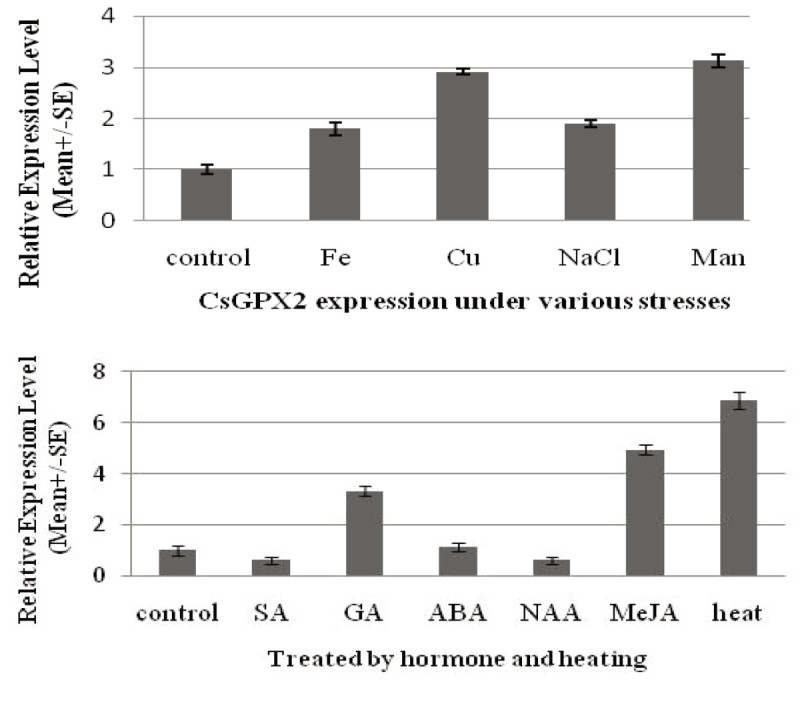
**The relative expression levels under abiotic and biotic stresses.** Intact seedlings roots were partly soaked in Fe (200 μM FeSO_4)_, Cu (200 μM CuSO_4_), 500 mM NaCl and Man (500 mM mannitol) for heavy metals, salinity and drought treatments. The seedlings were kept at 40°C for 3 hours for heat treatment. For plant hormones treatments, seedlings were with 1 mM SA, ABA, GA, NAA and MeJA for 12 hours. The control was soaked in or sprayed only deionized water and 18S rRNA was used as an internal control gene.

To detect the gene expression profiles under biotic stress, seedlings were treated with starved tea geometrids, and the transcript levels were investigated by real-time qRT-PCR. Compared to the control, the gene relative expression level was 1.12, 1.14, 0.94, 1.17, 1.15 and 1.11 respectively, after insect feeding for 6, 12, 24, 48, 72 and 96 hours. This result showed gene expression had no significant up-regulation or down-regulation, which indicated that *Cs* GPX2 was not induced by herbivore damage (Figure 4). Real time qRT-PCR was applied to analyze the gene expression levels under abiotic treatments of heavy metals, plant hormones, heat, drought and salt stress. The *Cs* GPX2 transcript levels increased 1.8-, 2.9-, 1.9- and 3.1-fold under Fe^2+^, Cu^2+^, NaCl and mannitol treatments for 6 hours. Under heat treatment at 40°C for 3 hours, the gene expression increased 6.9-fold significantly. After hormone treatments for 12 hours, *Cs* GPX2 was induced by GA and MeJA with 3.3- and 4.9-fold increase respectively, but the gene transcription was not sensitive to SA, ABA and NAA stresses (Figure 5).

The *Cs* GPX2 expression was induced by abiotic stresses including salinity, heavy metal toxicity, drought and heat. While it was only induced dramatically by hormones of GA and MeJA, increased a little under ABA treatment, and even decreased a little under SA and NAA treatment. That suggested *Cs* GPX2 expression level was more sensitive to heat, drought, heavy metal toxicity, but selectively to plant hormones with GA and MeJA. This result suggested that *Cs* GPX2 could potentially function in protecting tea plant from oxidative damage under some abiotic stresses. Because *Cs* GPX2 was not activated obviously under herbivore damage, the gene might have no function in biotic stress defense.

## Conclusion

Glutathione peroxidase (GPX) is the general name for a family of isozymes that removes ROS using GSH as an electron donor (Margis et al. 2008; Wang et al. 2012). Generally, in plants GPXs localized at different cell organelles as mitochondria, chloroplast, endoplasmic reticulum/cytosol or secreted (Ramos et al. 2009). The major two functions of GPXs in plants were to protect cell membranes from peroxidative damage and involved in redox transduction under stress (Gueta-Dahan et al., 1997; Miao et al. 2006; Gill and Tuteja 2010; Suzuki et al. 2011). When treated with stresses including salinity, heavy metal toxicity, drought, heat, cold and hormone, the expression of many GPX genes were enhanced dramatically (Holland et al. 1993; Avsian-Kretchmer et al. 2004; Ramos et al. 2009; Chang et al. 2009; Faltin et al. 2010).

In this paper, we successfully cloned a new gene designated as *Cs* GPX2 encoding glutathione peroxidase from tea plant. Bioinformatics analysis showed that *Cs* GPX2 contained the highly conserved metal ion-binding motif of ILAFPCNQF, and the amino acid sequence had high homology with GPXs of plants (Rodríguez Milla et al. 2003; Ramos et al. 2009). The *Cs* GPX2 expression was induced by abiotic stresses including salinity, heavy metal toxicity, drought and heat, and similar results were observed in *Arabidopsis* and *Citrus* (Holland et al. 1993; Rodríguez Milla et al. 2003; Miao et al. 2006). The gene expression were only induced dramatically by hormones of GA and MeJA, increased a little under ABA treatment, and even decreased a little under SA and NAA treatment, which suggested that the gene expression was more sensitive to heat, drought, heavy metal toxicity, but selectively to plant hormones with GA and MeJA. Furthermore, because of *Cs* GPX2 being not activated obviously under herbivore damage, the gene might have no function in biotic stress defense. Because those abiotic stresses lead to ROS production and *Cs* GPX2 was significantly induced, which indicated that *Cs* GPX2 may have potential function in protecting tea plant from peroxidative damage under abiotic stress.

In plants, most signal transduction pathways related to stress tolerance were divided into two types of dependent on ABA or ABA-independent (Shinozaki and Yamaguchi-Shinozaki 1996). The result showed *Cs* GPX2 was not induced by ABA, which suggested *Cs* GPX2 was an ABA-independent gene in stress signal transduction of tea plant. The expression profiles showed *Cs* GPX2 was ubiquitous in tea plant organs and regulated by different abiotic stresses, which indicated this gene contributed to the defense against oxidative damage caused by normal plant metabolism (Rodríguez Milla et al. 2003). These findings might help us to understand the high tolerance to heavy metals toxicity, mechanism of drought response and defense signal transduction way in tea plant.

## Authors’ original submitted files for images

Below are the links to the authors’ original submitted files for images. Authors’ original file for figure 1 Authors’ original file for figure 2 Authors’ original file for figure 3 Authors’ original file for figure 4 Authors’ original file for figure 5
