# Supplementary figures and images for: Cloning of a new glutathione peroxidase gene from tea plant (Camellia sinensis) and expression analysis under biotic and abiotic stresses
Source: Bot Stud. 2014 Jan 18;55:7. doi: 10.1186/1999-3110-55-7 (PMC5432830; doi:10.1186/1999-3110-55-7)

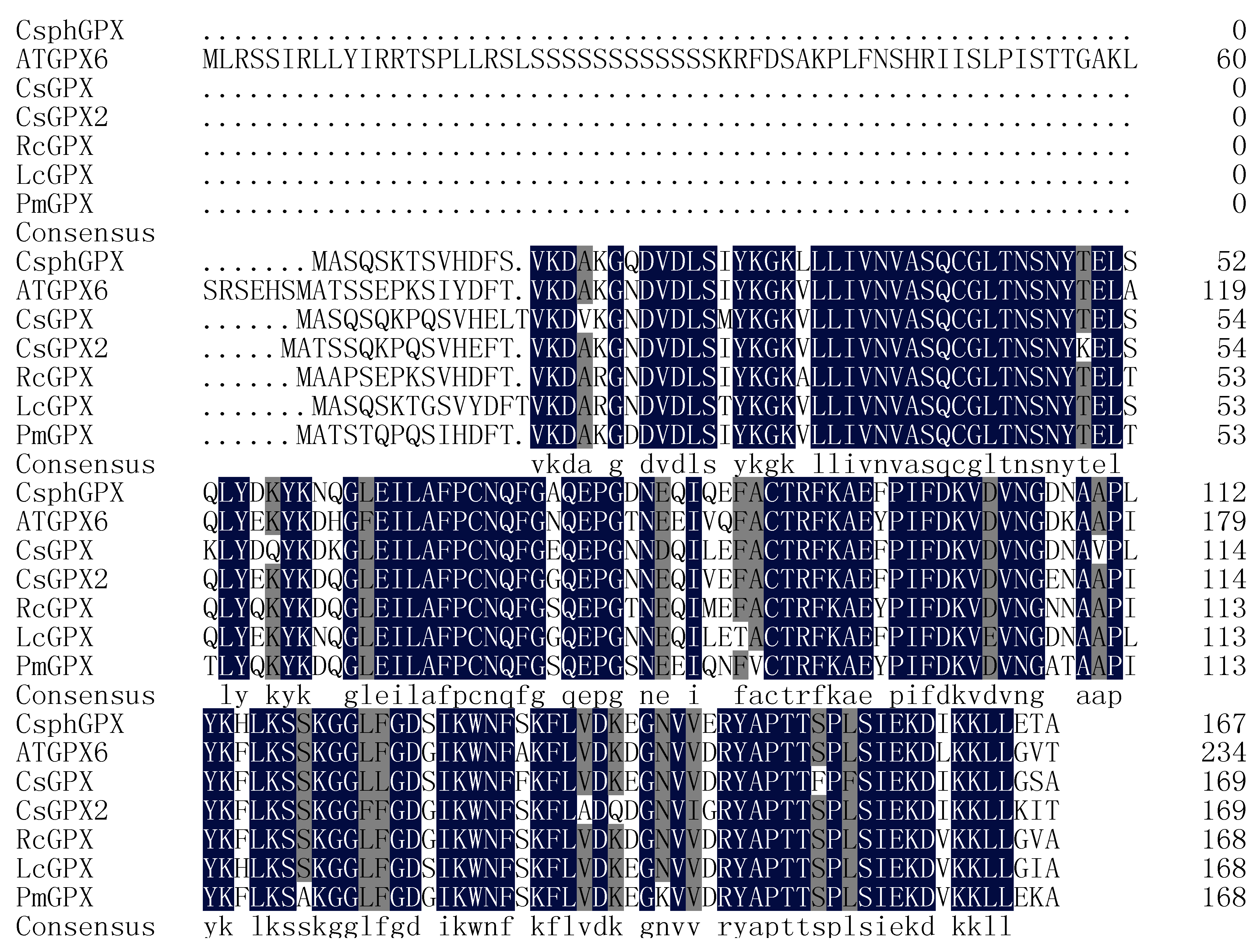

Supplement: Supplementary file 1 — Authors’ original file for figure 1 [file 40529_2013_57_MOESM1_ESM.tiff]

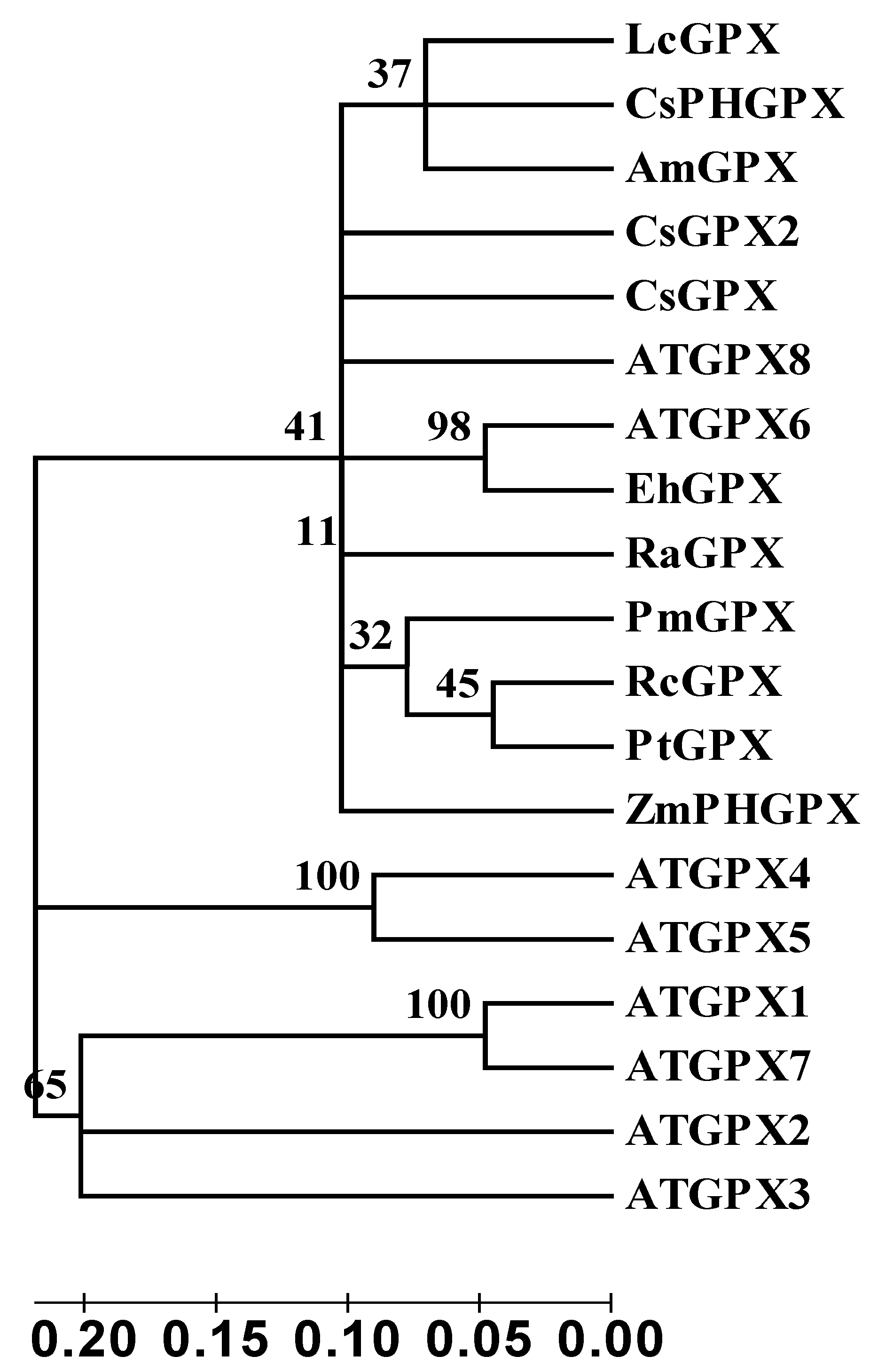

Supplement: Supplementary file 2 — Authors’ original file for figure 2 [file 40529_2013_57_MOESM2_ESM.tiff]

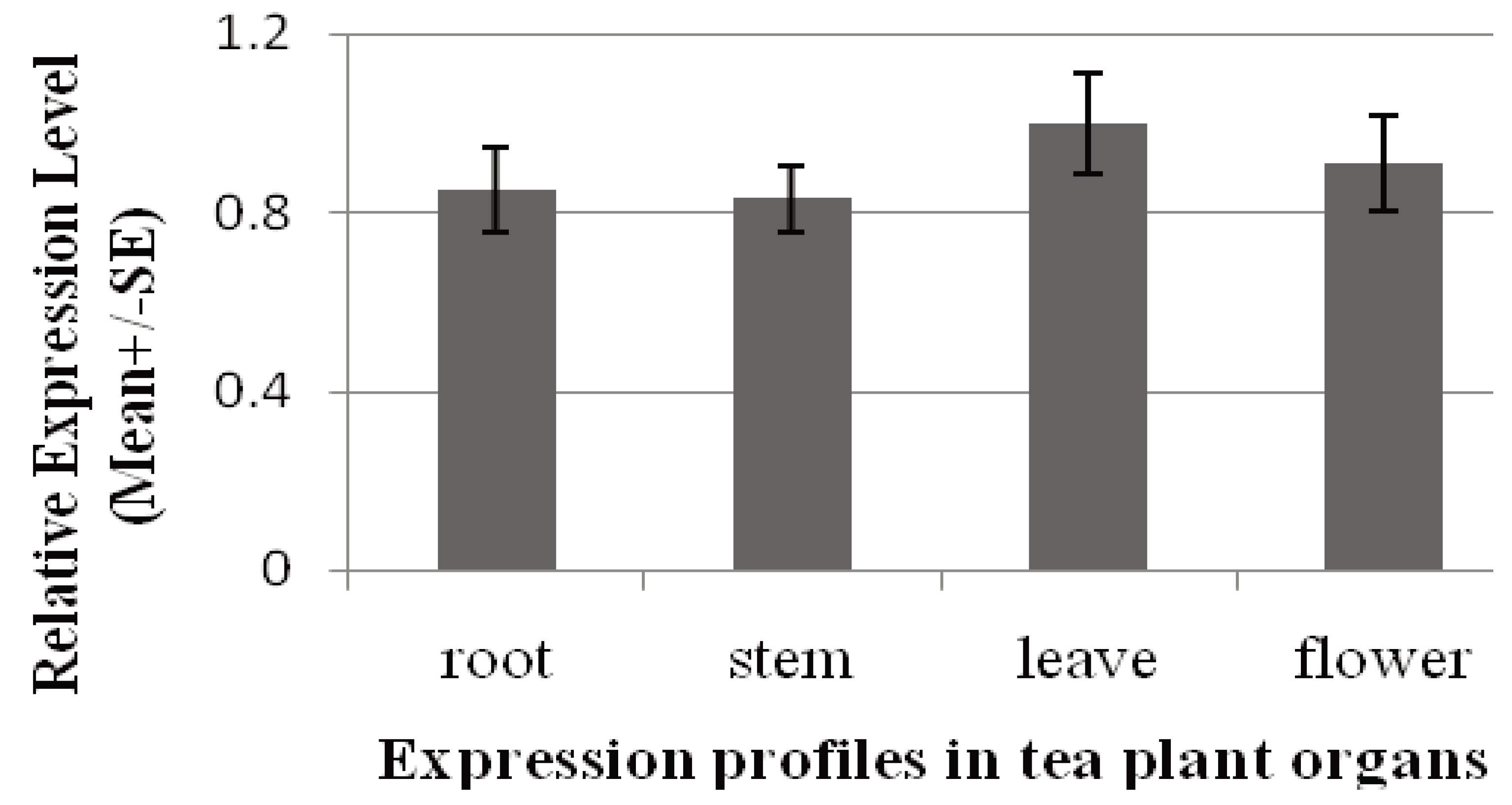

Supplement: Supplementary file 3 — Authors’ original file for figure 3 [file 40529_2013_57_MOESM3_ESM.tiff]

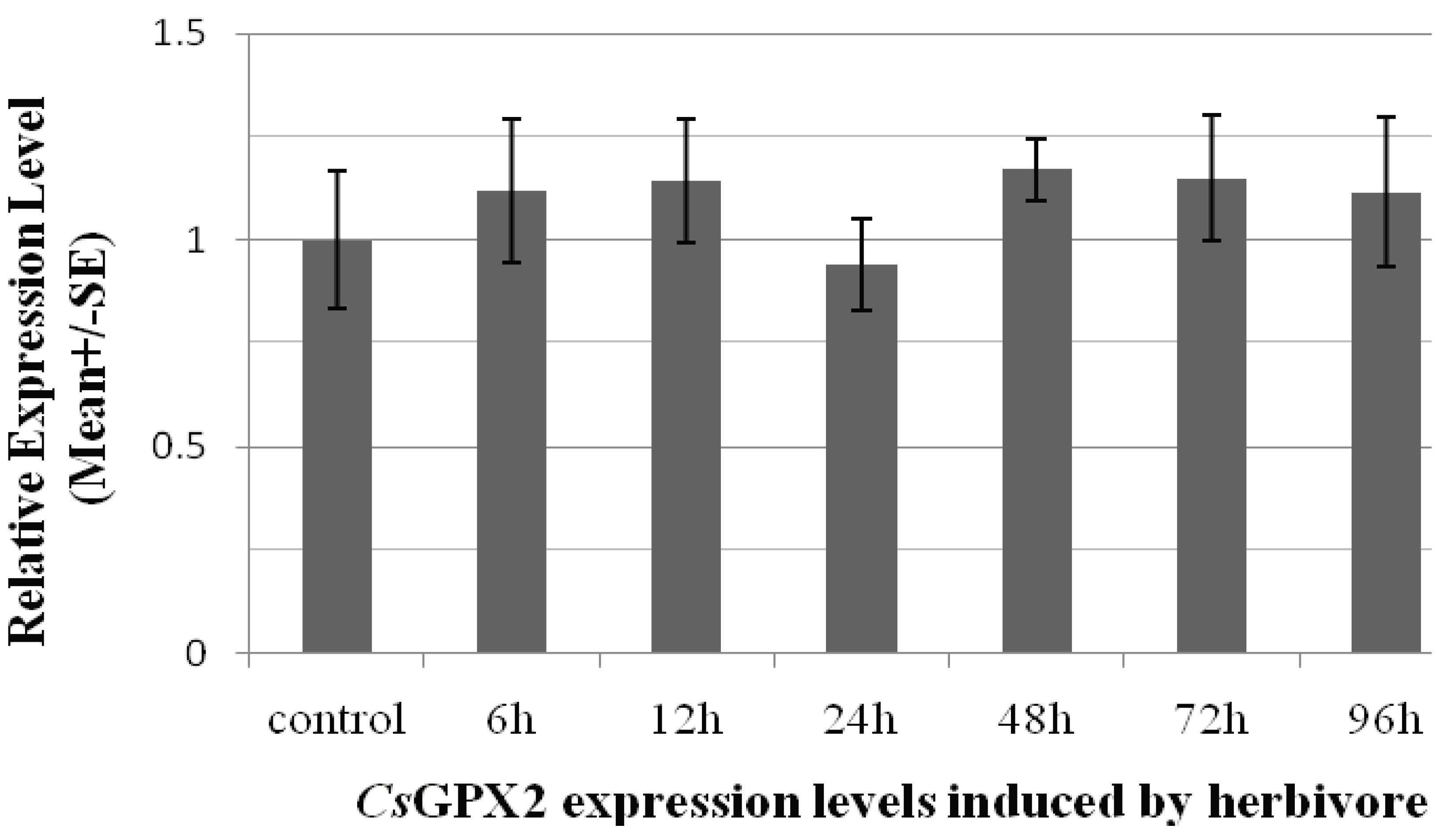

Supplement: Supplementary file 4 — Authors’ original file for figure 4 [file 40529_2013_57_MOESM4_ESM.tiff]

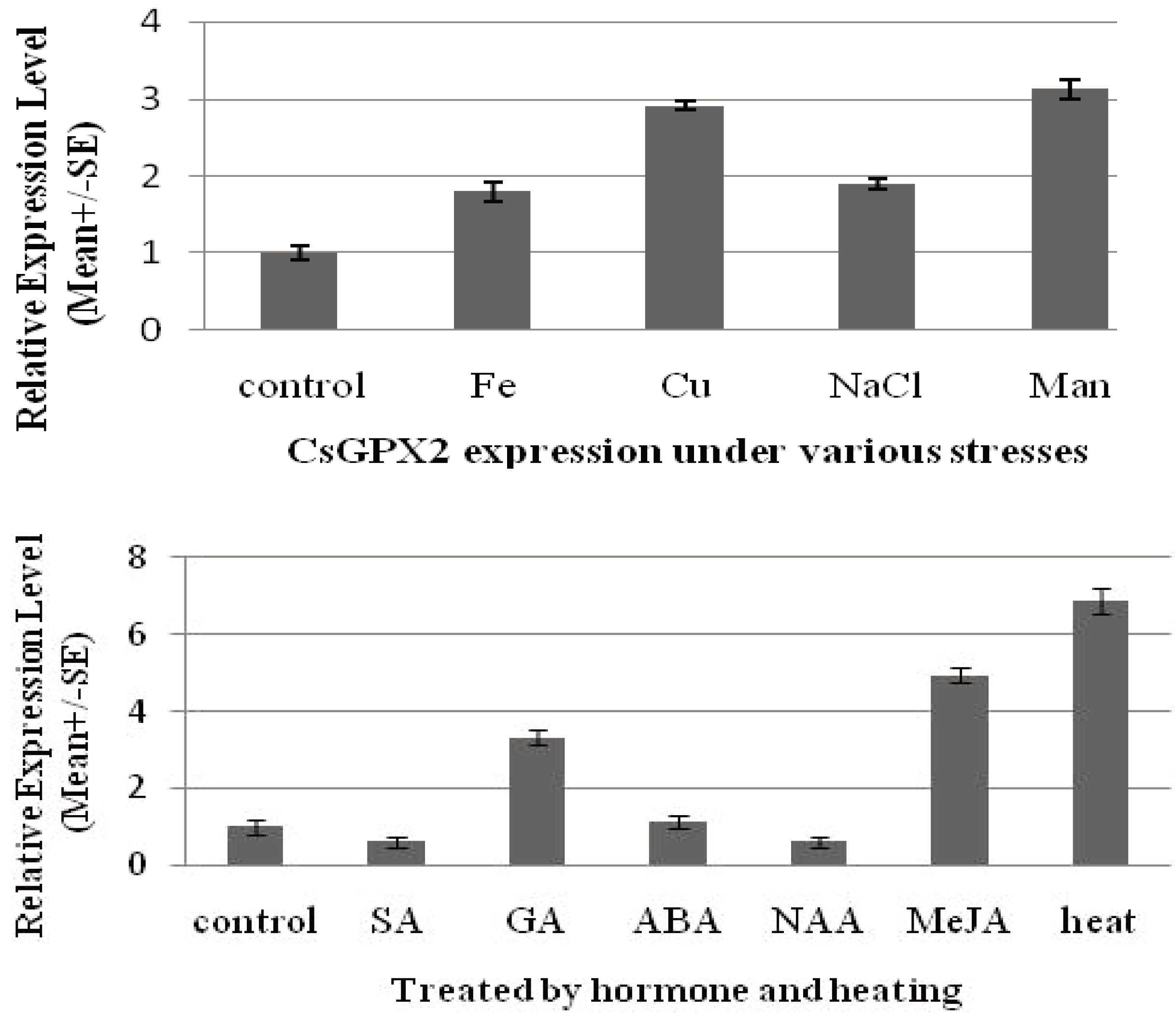

Supplement: Supplementary file 5 — Authors’ original file for figure 5 [file 40529_2013_57_MOESM5_ESM.tiff]
